# Behavioral determinants of immunization service utilization in Ethiopia: a cross-sectional community-based survey

**DOI:** 10.11604/pamj.supp.2017.27.2.10635

**Published:** 2017-06-09

**Authors:** Yohannes Ababu, Fiona Braka, Aschalew Teka, Kinde Getachew, Tefera Tadesse, Yohannes Michael, Zewdie Birhanu, Peter Nsubuga, Tersit Assefa, Kathleen Gallagher

**Affiliations:** 1 Immunization program, World Health Organization, Addis Ababa, Ethiopia; 2 Jimma University, Ethiopia; 3 Manuscript Writing Consultant, WHO, Ethiopia; 4 Immunization program , UNICEF, Ethiopia

**Keywords:** Behavior, knowledge, approval, intention, pentavalent vaccine, doses, advocacy

## Abstract

**Introduction:**

According to the Ethiopian Health Sector Development Plan IV annual performance report (HSDP IV), Ethiopia targeted to reach 90% coverage with DPT-Hib-HepB 3 (Pentavalent3) vaccine and 86% coverage with measles vaccine in 2010- 2011. However, the actual performance fell-short of the intended targets due to several reasons. Therefore, a nationwide comprehensive study was conducted to examine the behavioral determinants of immunization practices in the Ethiopian context. The study employed the Modified Steps of Behavioral Change (SBC) Model as a theoretical lens.

**Methods:**

A cross-sectional study was conducted in May 2012 in all the nine regions and the two city administrations of Ethiopia. The study used a community-based quantitative survey design comprising of multistage cluster sampling to draw relevant data from a sample of 2,328 caretakers whose children were 12-23 months of age at the time of data collection.

**Results:**

Overall, the multivariate analysis findings revealed that caretakers, who had high knowledge were 2.24 times more likely to vaccinate their children than participants had low knowledge (OR= 2.24, 95%CI: 1.68-2.98). Participants who had high approval were 2.45 times more likely to vaccinate their children than participants who had unfavorable approval (OR= 2.45, 95%CI: 1.67-3.59); and participants who had high intention were 6.49 times more likely to vaccinate their children with pentavalent3 vaccines than participants who had low intention(OR= 6.49, 95%CI: 4.83-8). Also, it was clear from the regression analysis that aspects of caretakers' demographic characteristics were significant predictors of their immunization practice for the sample group.

**Conclusion:**

We identified that caretakers' knowledge, approval, intention, parents' residence, and religious backgrounds were associated with immunization service utilization. To achieve sustainable behavioral change on immunization service utilization of the caretakers in Ethiopia, this study suggests investing in activities that enhance caretakers' knowledge, approval, intention, and practice components represented in the behavioral change model.

## Introduction

Immunization is one of the most cost-effective public health interventions to curb potential health problems globally. As of 2011, the World Health Organization (WHO) estimated that immunization averted 2-3 million deaths globally. In Ethiopia, from 1960-2002, a 50% reduction in under-5 mortality was observed and the immunization program saved the lives of nearly 4 million children [1, 2]. However, several studies reveal that millions of people have still not benefited from the protection that vaccination provides and remain at risk of life-threatening illnesses every day. For instance, there are large numbers of unvaccinated children in Ethiopia [3]. According to the Ethiopian Health Sector Development Plan IV (HSDP IV) annual performance report of 2011, the country aimed to reach 90% coverage with DPT-Hib-HepB 3 (Pentavalent3) vaccine and 86% coverage with measles vaccine in 2010 and 2011. However, the actual performance fell short of the target and DPT-Hib-HepB 3 and measles vaccine coverage dropped from 86.0% to 84.7% and 82.4% to 81.5% respectively from 2010 to 2011. A substantial decline in Pentavalent3 vaccine coverage was observed in Afar, Oromiya, Somali, and Harari regions. The 2010 and 2011 surveillance reports also indicated that 38,288 suspected measles cases and 182 deaths were reported from all regions; the major reasons for the outbreaks were low immunization coverage [4] We conducted a study to identify the potential determinants of immunization service utilization by caretakers from a broader perspective using the Modified Process of Steps of Behavioral Change (SBC) Model [5]. The primary purpose of the study was to investigate the potential behavioral and socio-economic determinants of immunization service utilization. We aimed to use the results of the study to suggest potential behavioral interventions that could help to improve immunization service utilization in Ethiopia.

## Methods

**Study area and period:** the community-based cross-sectional study was conducted in May 2012 in all the nine regions and the two city administrations of Ethiopia.

**Sample size and sampling method:** the study population for the community survey consisted of children aged 12-23 months. We used the immunization coverage survey standard formula recommended by World Health Organization to calculate the sample size for each region; we used Epi Info statistical software (Centers for Disease Control and Prevention, Atlanta, USA) with the following formula:

**N= DE (Zα/2)^2^ P (1-P)/d^2^** where:

DE=3 (design effect from the multistage sampling technique employed in this study); P= Regional pentavalent3 coverage data; Zα/2 = 1.96 (The z-score corresponding to 5% level of significance or 95% confidence interval); and d= Margin of error (10%).

The total sample size was 2,328 caretakers. We selected study participants using multi stage cluster sampling. We determined the number of clusters per region based on the total number of children to be sampled in each region using the WHO Expanded Program on Immunization (EPI) survey recommendations, previous studies and availability of sampled children per cluster in each region [6].

**Survey instrument:** primary data were collected using a standardized pretested questionnaire which had structured and semi-structured questions. The questionnaire covered caretakers' socio-demographic variables. It also included aspects to assess caretakers' knowledge, intention, approval, practice and advocacy on immunization service utilization, as well as availability of communication devices (such as television, mobile phone, and radio) and sources of information regarding immunization services. The English version of the questionnaire was translated to local languages, including Amharic, Somaligna, Afan Oromo, Tigrigna and Afarigna.

**Figure 1:**
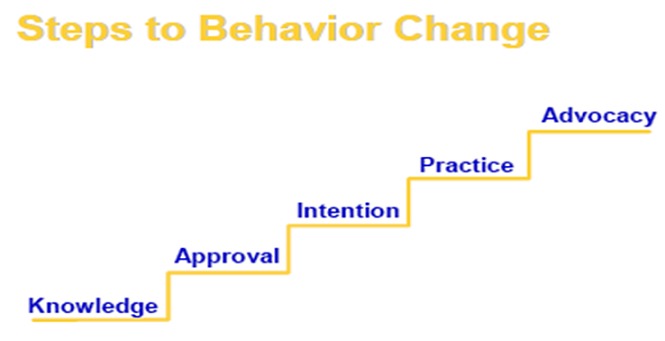
Diagrammatic representation of the conceptual framework for behavioral change (SBC) model

**Measures and scoring procedures:** knowledge: was measured using composite score of 11 items. We calculated participants' score out of 11 and converted the score into a percentage. If participants got a score of 60% and above, they would be classified as being knowledgeable and, classified as being not knowledgeable if they got a score less than of 60% . Approval: was measured using composite score of five items. The overall score for a respondent was summed and converted to a percentage. Accordingly, those participants who scored 60% and above were categorized as favorably approved the use of vaccination, while participants who scored less than 60% were categorized as lacking approval of vaccination. Intention: was measured using composite score of five items. We calculated participants' score out of 5 and converted the score to percentage .Those participants who scored 60% and above were categorized as having an intention to immunize their children and participants who scored below 60% were categorized as having no intention to immunize their children. Practice: was determined based on whether or not the child received the pentavalent3 vaccine from the card or history or certificate. Advocacy: was measured with six items. If a respondent responded positively, he or she received a point which was totaled out of six and converted to a percentage. Those participants who scored 60% and above on overall advocacy score were categorized as advocates of the immunization program and participants who scored less than 60% were considered as having less advocacy inclination of immunization service to others. Behavioral change process: This was determined in two ways. First, the levels of knowledge, approval, intention and advocacy were computed without restrictions to any requirements in moving from one stage of change to the next stage. Secondly, to determine the percentage of participants who had gone through the steps of change according to the recommendations of the Social Behavioral Change Model, participants who moved to the next stage of change without fulfilling the prerequisite stage (e.g., approval without knowledge, having intention without approving) were excluded at each stage. We referred this stage of change as “adjusted to the model” (Figure 1).

**Operational definitions: cluster:** was considered as kebele (the smallest administrative unit in Ethiopia). Urban residence: An administrative town with municipality service. Rural residence: A kebele which does not fulfill urban criteria and officially registered as rural kebele. Pastoralist residence: Pastoralist refers to subsistence practice in which people care for and domesticate animals such as camels, sheep and goats. In this survey, such areas were considered as pastoralist residences when officially recognized.

**Study variables:** dependent variable was utilization of immunization service based on pentavalent3 (DPT-Hib-HepB) vaccination status. Independent variables were socio-demographic variables, behavioral dimensions and source of information about immunization service.

Data collection procedures The data were collected by trained data collectors, who could speak local languages and had experience in survey undertaking. The data collectors were supervised by 14 supervisors for the 11 regions. Initially, the data collectors went to the center of the village and selected the direction by lottery method to obtain a random start direction, thereafter the data collector followed the direction to search for eligible households which contained children whose ages were 12-23 months.

**Figure 2:**
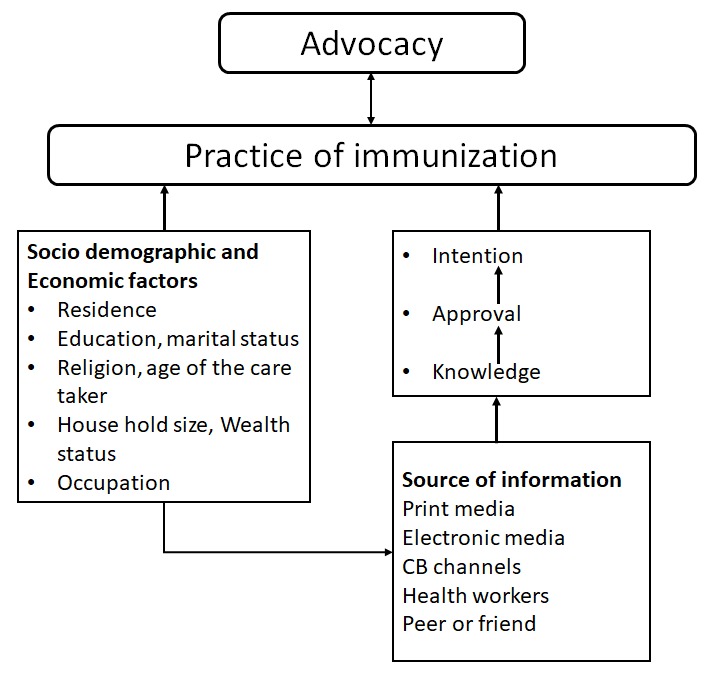
Diagrammatic representation of the modified conceptual framework of the behavioral determinant survey

**Data analysis:** the quantitative data were cleaned and entered into computer using SPSS version 17. Univarate analysis was used to describe the findings. Bivariate and multivariate logistic regression analyses were conducted to analyze the associations that existed among constructs. Odds ratios, confidence intervals (CI) at 0.1 alpha levels were used to adjust and identify the factors that were associated with immunization service utilization (Figure 2).

**Ethical considerations:** the study was approved by the Ethiopian Ministry of Science and Technology Ethical Review Board (Reference No: 310/622/04). Before the conduct of the field work, the researchers sought permission from each administrator at all levels. Each study participant was asked to participate in the study after explaining the aim of the study and after assuring confidentiality of personal information using code numbers instead of names.

## Results

There was a 100% response rate, of which, the large majority of them 2,174 (96.3%) were female. A total of 1,378 (60.7%) the study participants resided in rural areas. Half of the study participants were Muslims 1,141 (50.3%), followed by Orthodox 812 (35.8%). Out of 2,268 participants, 2,093(93.2%) were married. Most participants 773 (34%) were in the 30-34 years age group, followed by the 25-29 years age group had 602 (26.5%) participants. At total of 1,119 (49.6%) participants could not read and write. Most participants were housewives and farmers who constituted 1,262 (55.9%) and 439 (19.4%) respectively. Out of 2,268 participants, 1,279 (58.0%) were middle economic income class (Table 1).

**Table 1:** Socio-demographic and economic characteristics of the participants for the behavioral determinants survey, 2012, Ethiopia

Characteristics		N(%)
**Gender**	Female	2174 (96.3)
	Male	84 (3.7)
**Income**	Poor	446 (20.2)
	Middle	1279 (58.0)
	High	480 (21.8)
**Religion**	Orthodox	812 (35.8)
	Muslim	1141 (50.3)
	Protestant	282 (12.4)
	Catholic	26 (1.1)
	Others	8 (0.4)
**Marital status**	Married	2093(92.3)
	Single	45 (2.0)
	Divorced	81 (3.6)
	Widowed	49 (2.2)
**Age**	15-19	67 (2.9)
	20-24	366 (16.1)
	25-29	602 (26.5)
	30-34	773 (34.0)
	35-39	269 (11.8)
	40-44	117 (5.1)
	45-49	38 (1.7)
	>50	40 (1.8)
**Education**	Cannot read and write	1119 (49.6)
	Read and write but no formal education	430 (19.1)
	Attended formal education	707 (31.3 )
**Occupation**	Housewife	1262 (55.9)
	Farmer	439 (19.4)
	Merchant	231 (10.2)
	Government employed	188 (8.3)
	Private employed	47 (2.1)
	Daily Laborer	60 (2.7)
	Other	31 (1.4)

Participants who owned mobile phones, radios, and televisions (TV) were 1,299 (57.2%), 1,249 (55.0%) and 706 (31%), respectively. The proportion of study participants who owned TV was lower in rural areas 149 (10%). The percentage of mobile phones distribution varied across regions; Addis Ababa participants had the highest 28, (87.5%) while Somali region had the lowest 92 (44.2%) (Table 2).

**Table 2:** Ownership of communication devices among the study participants by region and residence, 2012, Ethiopia

Region	Mobile phone N (%)	Radio N (%)	TV N (%)
Addis Ababa	28(87.5)	32(100)	30(93.8)
Afar	154(58.1)	204(77.9)	103(39.0)
Amhara	174 (64.7)	133(49.3)	94(35.1)
Benishangul G.	173 (54.6)	158(49.7)	72 (22.6)
Dire Dawa	113(54.6)	109(52.9)	115(55.8)
Gambella	147 (56.3)	61(23.4)	22(8.4)
Harari	129(65.5)	124(64.2)	118(61.1)
Oromiya	135 (56.2)	145 (60.7)	44(18.6)
SNNPR	81( 47.6)	97(57.4)	36(21.2)
Somali	92(44.2)	133 (67.2)	41(20.0)
Tigray	67 (69.8)	38(39.2)	25(25.8)
**Residence**			
Urban	687 (77.3)	549 (62.5)	551 (62.5)
Rural	606 (44.1)	685 (50.1)	149 (10.9)
Average	1299 (57.2)	1249 (55.0)	706 (31.1)

The urban participants obtained immunization information from TVs, health workers and radio, which constituted 539 (60.6%), 514 (57.8%) and 512 (57.5%) respectively. Rural residents obtained information from health workers, radio and town criers, accounting for 766 (55.9%), 580 (42.3%) and 485 (35.4%) respectively. Religious leaders as immunization sources of information in Afar region and Addis Ababa contributed 23% and 0% respectively (Table 3).

**Table 3:** Sources of information about immunization among the study participants by region and residence, 2012, Ethiopia

Region	Radio	TV	Kebele	Peer	HWs	HEWs	Criers	Clan	Religiou
	N(%)	N(%)	N(%)	N(%)	N(%)	N(%)	N(%)	N(%)	N(%)
**Addis Ab**	20 (62.5)	25 (78.1)	2 (6.2)	0(0)	12 (37.5)	0(0)	1 (3.1)	0(0)	0(0)
**Afar**	194(73.5)	118(44.7)	120 (45.5)	90 (34.1)	115 (43.6)	82 (31.1)	46 (17.4)	48 (18.2)	61 (23.1)
**Amhara**	22(45.2)	104 (38.5)	33 (12.2)	29 (10.7)	154 (57.0)	39 (14.4)	158(58.5)	26 (9.6)	7 (2.6)
**BenisG**	34(42.1)	85(26.7)	87 (27.4)	84 (26.4)	248 (78.0)	75 (23.6)	130(40.9)	75 (23.6)	27(8.5)
**DireDawa**	108(52.2)	102(49.3)	62 (30.0)	52 (25.1)	131 (63.3)	21(10.1)	106(51.2)	70 (33.8)	3 (1.4)
**Gamb**	73(28.2)	30 (11.6)	62(23.9)	42 (16.2)	201 (77.6)	94 (36.3)	135(52.1)	84(32.4)	51 (19.7)
**Harari**	127(64.5	118(59.9)	39 (19.8)	13 (6.6)	88 (44.7)	74 (37.6)	85 (43.1)	12(6.1)	3(1.5)
**Oromiy**	123(51.2)	44 (18.3)	41 (17.1)	29 (12.1)	81 (33.8)	144(60.0)	33 (13.8)	22 (9.2)	17 (7.1)
**SNNPR**	70 (41.2)	40 (23.5)	22 (12.9)	15 (8.9)	102 (60.0)	42 (24.7)	110(64.7)	29 (17.1)	22 (12.9)
**Somali**	96 (46.6)	19(9.2)	40 (19.4)	10 (4.9)	78 (37.9)	71 (34.5)	32 (15.6)	0 (0.00)	3 (1.5)
**Tigray**	25(25.5 )	24 (24.7)	16 (16.5)	11 (11.3)	70 (72.2)	48 (49.5)	0(0)	8 (8.2)	4 (4.1)
**Residenc**									
**Urban**	12(57.5)	539 (60.6)	239 (26.9)	147(16.6)	514 (57.8)	205(23.0)	321(36.1)	167(18.8)	54 (6.1)
**Rural**	580 ( 2.3)	170 (12.4)	285 (20.8)	228(16.6)	766 (55.9)	485(35.4)	515(37.6)	207(15.1)	144(10.5)

As shown in Table 4, the results of the adjusted model revealed that the participants who have adequate knowledge about immunization constituted 76.7% of the total sample. Similarly, the percentage of participants who approved immunization service importance accounted for the 72.3%; and participants who had an intention to use immunization service was 67.6%. A total of 60.0% of the participants vaccinated their children with having adequate knowledge, approval and intention. Only 27 (8%) of the participants advocated for the immunization program by having adequate knowledge, approving the program, having intention and practice by oneself (Table 4).

**Table 4:** Behavioral level of the stage of change process among study participants, 2012, Ethiopia

Behavioural stage Variables	* Unadjusted model (N=2328)	** Adjusted model (N=2328)
Knowledge		
Good knowledge	76.7%	76.7%
Approval		
Favourably Approved	89.8%	72.3%
Intention Positively Intended	88.7%	67.6%
Practice Penta 3 coverage	73.9%	60%
Advocacy positively Advocate	40.9%	27.8%

* unadjusted value shows that the participants levels of stage of change without restriction by the Behavioral Change Theoretical model

** Adjusted value indicates that each stages of the participants stage of change process that fit with stage of Behavioral Change Theoretical model

In the bivariate analysis, the odd of older participants vaccinating their children was 3% less than for younger participants. The odds of Rural dwellers and Pastoralist community participants vaccinating their children were 48% and 53% less than from urban residents participants (OR= 0.52, 95% CI: 0.42-0.64) and (OR= 0.47, 95% CI: 0.42-0.64), respectively. The odds of could read and write but with no formal education and couldn't read and write participants vaccinating their children were 54% and 47% less than form formal education participants (OR= 0.46, 95% CI: 0.37-0.58) and (OR= 0.53, 95% CI: 0.40-0.71), respectively. The odd of Muslims participants vaccinating their children was 44% less than form Orthodox participants (OR= 0.56, 95% CI: 0.45-0.69).

The odds of high level of knowledge of immunization participants vaccinating their children was 40% higher than from lower knowledge participants (OR= 3.40, 95% CI: 2.71-4.28). The odds of high of approval for immunization participants vaccinating their children was 70 % higher than had unfavorable approval participants (OR= 3.70, 95% CI: 2.76-4.89); and the odds of had high intention for immunization participants vaccinating their children with pentavalent3 vaccines was 70% higher than had low intention participants (OR= 3.70, 95% CI: 2.83-4.02) (Table 5).

**Table 5:** Distribution of bivariate and multivariate logistic regression analysis of overall variables by pent3 coverage, 2012, Ethiopia

Variables	Unadjusted		Adjusted	
	OR	CI	OR	CI
				
**Age**	0.97	(0.96-0.99)	0.99	(0.97-1.00)
			>	
**Residence**				
Urban*	1		1	
Rural	0.52	(0.43-.64)	0.44	(0.32-.60)
Pastoralist	0.47	(0.28-.80)	0.54	(0.25-1.13)
**Education**				
Formal educated*	1		1	
Can read and write	0.46	(0.37-.58)	0.64	(0.39-.77)
Can’t read and write	0.53	(0.40-0.71)	0.5	(0.38-.76)
**Income**				
Low*	1		1	
Middle	2.09	(1.54-2.83)	1.05	(0.72-1.53)
High	1.37	(1.09-1.72)	0.95	(0.64-1.48)
**Religion**				
Orthodox*	1		1	
Muslim	0.56	(0.45-0.69)	0.85	(0.63-1.14)
Protestant	1.41	(0.98-2.02)	2.14	(1.28-3.59)
Catholic	0.89	(0.35-2.25)	1.07	(0.31-3.64)
Others	0.8	(0.16-4.00)	1.41	(0.15-18.94)
**Knowledge**				
Low*	1		1	
High	3.41	(2.71-4.28)	2.24	(1.68-2.98)
**Approval**				
Low*	1		1	
High	3.68	(2.76-4.89)	2.452	(1.67-3.59)
**Intention**				
Low*	1		1	
High	3.68	(2.83-4.02)	6.49	(4.83-8.71)

* Reference Category

A multiple logistic regression was performed to ascertain the effects of age, residence, education income, and religion, knowledge of immunization, approval and intention of immunization, which had statistically significant relationships in the bivariate analysis on the likelihood that children received pentavalent3 vaccines.

The results of multiple regression analysis for the adjusted model revealed that rural dwellers were 0.44 times less likely to vaccinate their children as compared to urban dwellers (OR= 0.44, 95% CI: 0.32-0.60); participants who were Muslim was 0.85 times less likely to vaccinate their children than participants who were Orthodox (OR= 0.85, 95% CI: 0.63-1.14) ; participants who could read and write but had no formal education and participants who couldn't read and write were 0.64 and 0.50 times less likely to vaccinate their children as compared to people who attended formal education (OR= 0.64, 95% CI: 0.39-0.77) and (OR= 0.50, 95% CI: 0.38-0.76). Participants who had high knowledge were 2.24 times more likely to vaccinate their children than participants had low knowledge (OR= 2.24, 95% CI: 1.68-2.98). Participants who had high approval were 2.45 times more likely to vaccinate their children than participants who had unfavorable approval (OR= 2.45, 95% CI: 1.67-3.59); and participants who had high intention were 6.49 times more likely to vaccinate their children with pentavalent 3 vaccines than participants who had low intention(OR= 6.49, 95% CI: 4.83-8.71) (Table 5).

## Discussion

Overall, there is a fairly good level of knowledge about immunization services demonstrated through our study (76.7%). This proportion is however lower than some previous studies conducted in Ethiopia and Nigeria [7, 8]. We also found that 72.30% of the participants had approved or had very favorable attitude towards immunization service utilization; this finding is almost consistent with studies conducted in Nigeria and Poland [7]. However, the proportion of participants who developed intention was 67.6%. This implies that some parents intended to vaccinate their children without actually approving immunization service. This might happen due to peer influence or imitation of other parents.

Immunization practice was below the national target; pentavalent3 vaccination coverage among the children sampled in the survey was 60%. This finding is consistent with other findings of studies in rural Ethiopia and Nigeria [8, 9]. However, even though the coverage was suboptimal, our study showed relatively higher level of immunization service utilization among the community compared to figures in the last Ethiopia Demographic Health Survey (EDHS) report that revealed penvalent 3 coverage of 35% [10]. The observed difference might be attributed to difference in design of the survey. Advocacy, the final step to behavior change, is a vital part of the process because it represents a level of commitment that goes beyond the mere practice of a new behavior. Our survey revealed that only 28% of the participants were found at the stage of advocacy, (i.e., expressed commitment to support immunization program in their community). Also, the communication approach may lack appropriate strategies to boost up people's confidence and to prepare them for advocacy.

We identified that good level of knowledge was associated with positive behavior of immunization service utilization, which is consistent with studies conducted in Nigeria and Ethiopia [11, 12]. Approval and intention were significantly associated with immunization practice and this finding is consistent with some earlier studies conducted in Ethiopia, Brazil and Nigeria [11, 13, 14]. In addition, we found out that caretakers' residence and religious backgrounds were associated with low immunization uptake which also reported in other countries particularly in Brazil, Uganda, India and Iran [14–19].

Although we revealed important findings on determinants of immunization services utilization in Ethiopia, the study did not address immunization service determinants like immunization service quality, logistic inventory, inter personal communication skills and practice of health workers. These determinants may have influences on caretakers' immunization service utilization and deserve investigation in the future.

## Conclusion

In conclusion we identified that caretakers' knowledge, approval, intention, parents' residence, and religious backgrounds were significantly associated with immunization service utilization. However, age, marital status, occupation and income had no association with immunization service utilization. Also communication channels like town criers /megaphone announcements; religious, clan and Kebele leaders were the most available communication channels at community level in Ethiopian context . However, we identified that there is poor utilization of traditional channels like towncriers , religious, clan and Kebele leaders to promote immunization service utilization across the studied regions. To achieve sustainable behavioral change in immunization service utilization in Ethiopia, we recommended to all immunization partners and stakeholders to pay special attention to promote activities that enhance care takers' knowledge, approval and intention components of the behavioral change process. A mix of communication channels (traditional and modern) including locally available communication channels to address illiterate parents, should be deployed to increase knowledge on immunization, and ultimately impact practice to increase coverage rates in Ethiopia.

### What is known about this topic

- Availability of immunization service and antigens determine immunization service coverage. Studies aimed to assess the knowledge and attitude of mothers attending antenatal clinic. Previous studies on immunization service utilization most often consider aspects of demographic characteristics of caretakers without including behavioral aspects and without examining how that have influenced caretakers immunization service utilization. Most studies on caretakers' immunization service utilization emphasize on inequities in coverage between and within countries;
- Social determinants have the potential to affect immunization service utilization in many parts of the world, with globalization and ease of communication leveraging the process. Research reveals different types of social determinants affecting immunization efforts in various countries;
- While it is common to link caretakers' immunization service utilization data with some demographic characteristics, there is minimal link created so far between immunization service utilization and behavioral components. Due to this, little is known about behavioral indicators and their potential influences on the caretakers' immunization practices.

### What this study adds

- This study provides a new perspective and a conceptual model of studying immunization service utilization considering the behavioral aspects of caretakers and examining how that have influenced caretakers immunization service utilization in the Ethiopian context;
- Improving caretakers immunization service utilization requires much more than the results of survey on their experiences. We also need to link immunization service utilization data with some demographic characteristics and personal behaviors so that we can understand which of these demographics and behavioral indicators have significant influence on the caretakers' immunization practices. By doing so, we'll surely discover some demographic characteristics and personal behaviors that are related with immunization service utilization;
- Above all, this study provides empirical evidence testifying the application of a behavioral model and the corresponding behavioral indicators that should be considered for effective behavioral change planning used to improve caretakers' immunization service utilization and behavioral change intervention in Ethiopian context.

## Competing interests

Authors declared they have no competing interests in this study. The views expressed in the perspective articles are those of the authors alone and do not necessarily represent the views, decisions or policies of the institutions with which they are affiliated and the position of World Health Organization.
